# High mortality co-infections of COVID-19 patients: mucormycosis and other fungal infections

**DOI:** 10.15190/d.2021.5

**Published:** 2021-03-31

**Authors:** Kinal Bhatt, Arjola Agolli, Mehrie H. Patel, Radhika Garimella, Madhuri Devi, Efrain Garcia, Harshad Amin, Carlos Domingue, Roberto Guerra Del Castillo, Marcos Sanchez-Gonzalez

**Affiliations:** ^1^Division of Clinical and Translational Research, Larkin Health System, South Miami, FL, USA; ^2^Pakistan Ziauddin Medical College, Karachi, Pakistan; ^3^Larkin Community Hospital, Hialeah, FL, USA

**Keywords:** SARS-CoV-2, COVID-19, mucormycosis, fungal infection, candida auris, aspergillosis, pneumocystis pneumonia, cryptococcus neoformans, coinfection.

## Abstract

Severe COVID-19 disease is associated with an increase in pro-inflammatory markers, such as IL-1, IL-6, and tumor necrosis alpha, less CD4 interferon-gamma expression, and fewer CD4 and CD8 cells, which increase the susceptibility to bacterial and fungal infections. One such opportunistic fungal infection is mucormycosis. Initially, it was debated whether a person taking immunosuppressants, such as corticosteroids, and monoclonal antibodies will be at higher risk for COVID-19 or whether the immunosuppresive state would cause a more severe COVID-19 disease. However, immunosuppressants are currently continued unless the patients are at greater risk of severe COVID-19 infection or are on high-dose corticosteroids therapy. As understood so far, COVID-19 infection may induce significant and persistent lymphopenia, which in turn increases the risk of opportunistic infections. It is also noted that 85% of the COVID-19 patients’ laboratory findings showed lymphopenia. This means that patients with severe COVID-19 have markedly lower absolute number of T lymphocytes, CD4+T and CD8+ T cells and, since the lymphocytes play a major role in maintaining the immune homeostasis, the patients with COVID-19 are highly susceptible to fungal co-infections. This report is intended to raise awareness of the importance of early detection and treatment of mucormycosis and other fungal diseases, such as candidiasis, SARS-CoV-2-associated pulmonary aspergillosis, pneumocystis pneumonia and cryptococcal disease, in COVID-19 patients, to reduce the risk of mortality.

## SUMMARY

*1. Introduction*

*2. COVID-19 and immunosuppression*

*3. Fungal co-infections in COVID-19*

- *3.1 Mucormycosis*

- *3.2 Candidiasis (Candida auris)*

- *3.3 SARS-CoV-2–assoc. pulmonary aspergillosis*

- *3.4 Pneumocystis pneumonia*

- *3.5 Cryptococcal disease (C. neoformans)*

*4. Current treatment options for fungal infections in COVID-19 patients*

- *4.1 Mucormycosis*

- *4.2 Candidiasis (Candida auris)*

- *4.3 SARS-CoV-2–assoc. pulmonary aspergillosis*

- *4.4 Pneumocystis pneumonia*

- *4.5 Cryptococcal disease (C. neoformans)*

*5. Discussion*

*6. Conclusion*

## 1. Introduction

Towards the end of 2019, an outbreak of a pneumonia-like infection occurred in Wuhan, China, and rapidly spread across the globe. With the utilization of genome sequencing technology, the disease was identified as coronavirus disease or more commonly known as coronavirus disease 2019 (COVID-19), and the etiological agent was identified as severe acute respiratory syndrome coronavirus 2 (SARS-CoV-2)^1^. As of March 2021, there are 93.3 million infected cases, with 2 million confirmed deaths reported worldwide. This respiratory illness has significantly impacted all sectors of life, primarily healthcare and the economy, and as a result, on March 11, 2020, the WHO officially recognized COVID-19 disease as a pandemic. The coronaviruses are enveloped, positive-sense single-stranded viruses that utilize the human angiotensin-converting enzyme 2 (ACE2) receptors located on cells in many organs/tissues, including the lung, heart, kidney, bladder, eyes, nasal and oral cavities, brain, thyroid, liver, gallbladder, stomach, pancreas, intestine, reproductive system of males and females, and skin, to gain entry and trigger a range of clinical manifestations^2^. Evaluating the data available from various parts of the world has demonstrated that the SARS-CoV-2 virus follows an exponential growth, and the mean basic reproduction number, which indicates the number of new infections that can arise from a single case of COVID-19 infection, is estimated to be 1.4-4^1^.

The COVID-19 disease is associated with a range of clinical manifestations, particularly due to SARS-CoV-2’s high human-to-human transmission rate via respiratory droplets^1^. However, the presence of the binding receptors in different tissues determines the range of clinical manifestations. The clinical manifestations range from a self-limiting illness with general symptoms, such as fever, cough, and headache, to more severe complications, including acute respiratory distress syndrome, sudden cardiac death, liver dysfunction, and multi-organ failure. An analysis of 13 studies on laboratory-confirmed COVID-19 cases conducted in China demonstrated the prevalence of COVID-19-associated co- infections with bacteria, viruses, and fungi^3^. Severe COVID-19 disease is associated with an increase in pro-inflammatory markers, such as IL-1, IL-6, IL-6, and tumor necrosis alpha, less CD4 interferon-gamma expression, and fewer CD4 and CD8 cells; this, therefore, increases the susceptibility to bacterial and fungal infections^4^. One such opportunistic fungal infection involved is mucormycosis. Mucormycosis is an opportunistic fungal infection that belongs to the zygomycete family and are ubiquitous in the environment. The major route of infection is via inhalations of spores, which then spread to the paranasal sinuses and lungs. Mucormycosis is non-pathologic in immunocompetent individuals as a result of the presence of an intact immunity via neutrophils which hence permit the elimination of these spores^5^. However, in immunocompromised patients such as those with uncontrolled diabetes mellitus, diabetic ketoacidosis, presence of an open wound, HIV/ AIDS, cancer, and organ transplant, mucormycosis can result in a severe invasive fungal infection^6^.

Infection with mucormycosis is associated with high mortality primarily due to complications such as cavernous sinus thrombosis, disseminated infection, osteomyelitis, and death. Diagnosis is made via routine blood work, biopsy, and radiological imaging. The standard protocol for the management is primarily reversal of risk factors, surgical debridement as well as intravenous antifungal medication such as Amphotericin B^6^. Infection with SARS-CoV-2 drastically impacts the immune system via induction of an inflammatory storm, an increase in neutrophil count as well a decrease in lymphocyte count, specifically CD4+ and CD8+ T cells. Neutrophils ensure the immunocompetence of individuals, so one would expect an even more increased immune response against mucormycosis fungi when reading that neutrophil numbers are rising during SARS-CoV-2. Consequently, these patients are at increased susceptibility of developing opportunistic infections such as mucormycosis, due to decreasing lymphocyte cells^5^. CD4+ and CD8+ T cells serve a prominent role against infection with mucormycosis via recruiting cytokines, such as IL-4, IL-10, IL-17, and IFN-γ^5^.

## 2. COVID-19 and immunosuppression

Initially it was debated whether a person taking immunosuppresants be at higher risk of getting COVID-19 or whether the immunosuppresive state would cause more severe COVID-19 infection^7^. However, currently the immunosuppressants are continued unless the patients are at greater risk of severe COVID-19 infection or are on high-dose corticosteroids therapy^8^. As understood so far, COVID-19 infection may induce significant and persistent lymphopenia which in turn increases the risk of opportunistic infections^9^. It is also noted that 85% of the COVID-19 patients’ laboratory findings showed lymphopenia^10^. This means patients with severe COVID-19 have markedley lower absolute number of T lymphocytes, CD4+T and CD8+ T cells^11^. And since the lymphocytes play a major role in maintaining the immune homeostasis^10^, the patients with COVID-19 are highly susceptible to fungal co-infections^8^. Moreover, the aggressive disease course of the SARS-CoV-2 virus damages the lung tissues and alveolo-interstitial lesions which puts the COVID-19 infected person prone to getting invasive fungal infections, specifically those that are airborne or with primarily pulmonary entry. These include infections like pneumocystis and mucormycosis^11^. Mucormycosis is a rare fungal infection which is often life-threatening^8^. It is characterized by vascular invasion by the fungal hyphae which leads to thrombosis and necrosis^8^. There has been a surge of mucormycosis co-infection in COVID-19 patients, many cases being reported worldwide.

## 3. Fungal co-infections in COVID-19

### 3.1 Mucormycosis**

Mucormycosis, previously known as zygomycosis, is a severe fungal infection caused by a group of molds called mucoromycetes. It can affect the sinuses, brain or lungs and therefore can be quite common in people suffering or recovering from COVID-19. The common symptoms linked to mucormycosis are swelling in one side of the face, fever, headache, nasal or sinus congestion, black lesions on nasal bridge or upper inside of mouth^12^. Fungi that most commonly cause mucormycosis are: Rhizopus species, Mucor species, Rhizomucor species, Syncephalastrum species, Cunninghamella Bertholletia, Apophysomyces species, and Lichtheimia species. Mucormycosis can present in many forms such as, gastrointestinal mucormycosis (more common in young children), rhinocerebral mucormycosis (more common in people with uncontrolled diabetes and in people who had a kidney transplant), disseminated mucormycosis, pulmonary mucormycosis (more common in people with cancer and in people who have had an organ transplant or a stem cell transplant) and cutaneous mucormycosis^13^.

Symptoms of some fungal diseases can be similar to those of COVID-19, including fever, cough, and shortness of breath.Laboratory testing is necessary to determine if a person has a fungal infection or COVID-19. Some patients can have COVID-19 and a fungal infection at the same time. People with severe COVID-19, such as those in an intensive care unit (ICU), are particularly vulnerable to bacterial and fungal infections. Mucormycosis and orbital compartment syndrome in addition to COVID-19, has been diagnosed in a previously healthy young female^14^. Other authors have reported cases of acute invasive fungal rhino-orbital mucormycosis^15^ and cavitary pulmonary mucormycosis^5,16^ in patients with COVID-19. Patients hospitalized for COVID-19 are at risk for healthcare-associated infections^17^, including candidemia, or bloodstream infections caused by Candida and other fungal diseases.

### 3.2 Candidiasis (Candida auris)

*Candida auris (C. auris)* is an emerging fungus that can cause outbreaks of severe infections in healthcare facilities and invasive candidiasis are some of the common presentations in COVID 19 patients. Clinicians should consider fungal pneumonias as a possible cause of respiratory illness, particularly if COVID-19 testing is negative. A total of 1,518 confirmed cases and 30 probable *C. Auris* cases, were reported by U.S. States as of October 2020^18^. A fungal infection commonly seen during COVID-19 is *C. Auris*. The main concern regarding this fungal infection is related to *C. auris* being a multi-drug resistant, being extremely hard to identify with standard laboratory methods and, *C. auris* having caused various outbreaks in healthcare settings. Some studies have suggested an increased risk for Candida species in COVID-19 patients resulting in poor outcomes^19,20^. The *C. auris* fungal infection is known to spread quickly in long-term care facilities. However, during the pandemic there have been an increasing number of reports of *C. auris* in COVID-19 acute care units. Researchers suspect that these outbreaks may be related to changes in routine infection control practices due to the health crisis. For example, there is a limited availability of gloves and gowns and there is a possibility of faulty cleaning and disinfection practices^21,22^. According to this the US Center for Disease Control (CDC) report, among 67 patients admitted to the COVID-19 unit and screened during point prevalence surveys, 35 (52%) received positive test results. Mean age of colonized patients was 69 years (range = 38–101 years) and 60% were male. Six (17%) colonized patients later had clinical cultures that grew *C. auris*. Among patients screened who had available medical records (17), two (10%) were admitted directly from a long-term care facility and eight (40%) died within 30 days of screening, but whether *C. auris* contributed to death is unknown^23^. A recent study showed that mortality among patients with COVID-19 and *C. auris* candidemia was of 83.3% even when the right antifungal treatment was used. All investigated isolates shown resitance to the amphotericin B^24^.

### 3.3 SARS-CoV-2–associated pulmonary asper-gillosis (CAPA)

*Aspergillus genera*, most frequently *Aspergillus fumigatus*, can be found almost everywhere in the environment and can be responsible for many infections in humans, including for the invasive pulmonary aspergillosis (IPA), chronic pulmonary aspergillosis (CAPA), allergic bronchopulmonary aspergillosis (ABPA), fungal asthma, aspergillus bronchitis or chronic rhinosinusitis. Invasive pulmonary aspergillosis, which is the most severe form of disease from *Aspergillus*, is known to be associated with high mortality rates. It is also a prominent complication seen in severely immunosuppressed individuals, including the indiviudals with hematopoietic transplantation, as well as those with structural lung damage treated with systemic corticosteroids for their underlying condition (e.g.chronic obstructive pulmonary diseases (COPD) patients). Once they occur, these super infections are associated with high mortality rates and may prolong the acute phase of COVID-19^25^. Among fungal co-infections in France, the incidence of putative invasive pulmonary aspergillosis was high (30%)^24,26^. To date, >100 cases of CAPA have been reported from many countries in Europe, Asia, Australia, and South America^27-29^, often occurring in patients with no other risk factor than COVID-19-associated ARDS^27^, and multiple of them proven by autopsy^28-30^. According to Hoenigl’s study, fungal diseases, and particularly CAPA, add insult to injury in a significant proportion of critically ill COVID-19 patients and are associated with high mortality rates, which may be reduced by early diagnosis and initiation of appropriate antifungal therapy. In the absence of antifungal prophylaxis, screening of COVID-19 ARDS patients for CAPA and other fungal diseases is essential^31^. CAPA should be suspected in severe COVID-19 patients who have worsening respiratory function or sepsis^18^.

When looking at incidences of CAPA in critically ill patients (e.g., patients on ICUs), rates vary substantially from 4% to as high as 35%. The challenges in diagnosing fungal infections, and particularly CAPA, are thought to explain this variety in incidence rates. Clinical symptoms and abnormalities observed when the chest is imaged are non-specific. Positive sputum or tracheal aspirates do not differentiate between colonization and infection, and to perform invasive diagnostic procedures (e.g. bronchoscopy and bronchoalveolar lavage or lung biopsy) is often not feasible due to the clinical state of the patient. Moreover, these procedures are risky due to the possible airborne transmission of SARS-CoV-2^32^.

Some studies show that as many as one third of the COVID-19 patients with severe disease that require intensive care may also be fighting another life-threatening infection: for example, invasive aspergillosis, a deadly fungal superinfection caused by Aspergillus mold^33^. During the retrospective observational study of COVID-19 patients, bacterial and fungal co-infections occurred in <5% of all cases. However, this is of significant concern due to their occurrence in the most vulnerable patients^34^.

### 3.4 Pneumocystis pneumonia

*Pneumocystispneumonia* also shares similar symptoms of COVID-19 such as fever, cough, difficulty breathing, chest pain, chills, fatigue, and coinfection with *Pneumocystis jirovecii* may not be appreciated in patients with severe SARS-CoV-2 infection. There have been reported cases of these two co-infections^35^. In another study, which included almost exclusively immunocompromised patients has reported an unexpectedly high proportion of critically ill COVID-19 patients detected with P. jirovecii (10/108 patients; 9.3%). COVID-19 patients mostly exhibited marked lymphopenia and alterations in lymphocyte functions, likely explaining the high-rate of *P. jirovecii* detection^36^. This association has been reported even in young severely ill patients^37^. Differentiating COVID-19 from *pneumocystis jirovecii* pneumonia is not usually possible from signs and symptoms. Sputum culture, RT-PCR, CT chest are recommended to diagnose on time and do a differential between COVID-19 and *P. jirovecii*^38^.

### 3.5 Cryptococcal disease (C. neoformans)

Disseminated *Cryptococcus neoformans (C. neoformans)* infection is a serious infection that can occur in immunocompromised patients. There have been reported cases of *C. neoformans* and COVID -19 patients. The case reported by Mohamad Y and colleges, highlights the importance of early suspicion of *C. neoformans *infection and other opportunistic infections in immunocompromised patients, putting in mind that patients with Cryptococci have a high risk of mortality within 30 days, which warrants the use of corticosteroid and immunomodulatory drugs in a critically ill patient with COVID‐19. In the current scenario, the use of immunosuppressive therapy should be justified and to be alert for opportunistic infection like *C. neoformans* infection, which can lead to sepsis and mortality^39^.

## **4. Current treatment options for fungal infections in COVID-19

### **4.1 Mucormycosis

Global guidelines have demonstrated detailed and different approaches for the diagnosis and management of mucormycosis in 2019 by the European Confederation of Medical Mycology (ECMM) and Mycoses Study Group Education and Research Consortium^40^. It states an immediate and complete surgical intervention should be taken in the first place. In addition to it, systemic antifungals should be added to first-line management. There is a strong recommendation of high dose liposomal amphotericin B along with the adequate dosage of intravenous isavuconazole and posaconazole. Both triazoles can be given as salvage treatment as well. In high-risk patients such as neutropenic patients who have graft versus host disease, prophylaxis with posaconazole can be given. There is not enough data on the usage of combinations of other antifungal drugs. Limited choice of therapy is creating significant pressure on patients with low incomes. There is an excellent margin for uncertainty, and so much research work can be on it^40^.

### 4.2 Candidiasis (Candia Auris)

Patients suspected or confirmed with *C. auris *should be treated with echinocandins (caspofungin, micafunging, and anidulafungin), azoles (fluconazole, voriconazaole, itraconazole), and Amophotericin B and its liposomes. Therapeutic drug monitoring for optimizing efficacy and limiting toxicity of azoles should be considered^40,41^. As this is often a multidrug-resistant candida infection, some strains may be resistant to all antifungals. A fungus highly likely to cause hospital setting outbreaks, requires urgent identification and treatment initiation with contact precautions in place^40,42^.

### 4.3 SARS-CoV-2–associated pulmonary aspergillosis (CAPA)

Drugs recommended for the treatment of CAPA are divided in two categories. For allergic aspergillosis and prevention, CDC recommends triazoles (itraconazole, voriconazole, posiconzaole, esaconzaole) with corticosteroids. However, for invasive aspergillosis, like the one seen in COVID-19, CDC recommends voriconazole, lipid amphotericin B formulations, posaconazole, isavuconazole, itraconazole, and echinococcus (micafungin or caspofungin). Triazoles can be a safe choice for patients, but therapeutic drug monitoring (TDM) is highly recommended, and the interaction between azoles drugs should be observed diligently to observe for side effects. If the patient develops aspergilloma, surgery may be required^40,43^.

### 4.4 Pneumocystis pneumonia

The standard treatment for the pneumocystis pneumonia is a combination of trimethoprim and sulfamethoxazole. Corticosteroids can also be used for the moderate to severe pneumonia with low oxygen levels^35-38^.

### 4.5 Cryptococcal disease (C. neoformans)

For asymptomatic patients and patients with mild-to-moderate pulmonary infections, CDC recommends fluconazole. For people with severe lung infections or infections in the central nervous system (brain and spinal cord), CDC recommended initial treatment with Amphotericin B in combination with flucytosine. Patient is then switched to fluconazole for an extended period of time until infection is clear. Dose and duration of the antifungal treatment may differ for pregnant women, children, and people with limited resources. Some patients may require surgery for removal of fungal growth, known as cryptococcomas^40,44^.

## 5. Discussion

Mucormycosis is associated with invasion of blood vessels, which results in ischemic necrosis. Mucormycosis has the potential to invade various systems in the body resulting in a myriad of clinical symptoms that progress rapidly. Based on the anatomic site involved, mucormycosis can be classified into the following forms: rhino-cerebral, pulmonary, gastrointestinal, cutaneous, and disseminated^8^. As a result of the harmful complications associated with opportunistic co-infections such as invasive fungal infections in COVID-19 disease, it is essential that health care workers, mainly physicians, are vigilant and aware of the possibility of infection and take the necessary precautions^45^.

Monte Junior et al. (2020) reported a case of an 86-year-old male patient with history of arterial hypertension, admitted to the emergency room with symptoms of acute diarrhea, cough, dyspnea, and fever that started 5 days ago with a confirmed COVID-19 diagnosis. Within 5 days of admission, patient’s hemoglobin level of 14.3 mg/dL dropped to level of 5.6 mg/dL, which required infusion of three units of red blood cells. Esophago-gastroduodenoscopy to rule out cause of internal bleeding revealed two giant gastric ulcers with necrotic debris, confirmed to be mucormycosis upon biopsy. This patient did not have any other typical comorbidities to suggest suspicion of mucormycosis, such as diabetes. A rare disease of gastrointestinal mucormycosis was contributed to immune dysregulation caused by COVID-19. Despite intensive care, this patient died within 36 hours of the procedure. When the anticipated gastrointestinal (GI) mucormycosis symptoms such as fever, nausea, abdominal pain, GI bleeding and perforation are missing, establishing a correct diagnosis early on is challenging. Since the GI mucormycosis is known to have up to 85% fatality rate, early diagnosis and treatment becomes imperative in patient’s survival^8^.

Mehta et al. (2020) presented a case of a 60-year-old male patient with a past medical history of >10 years of diabetes with a three-day history of severe breathlessness, pyrexia, tachypnea and generalized malaise. While admitted for COVID-19 management, on day 10, patient developed bilateral lid edema with right eye prominence. MRI of the brain showed soft tissue swelling of orbits and paranasal sinuses with mucosal thickening. Patient was initially diagnosed with orbital cellulitis and ophthalmic consultation was obtained the next day. Upon examination, the ophthalmologist suspected a diagnosis of invasive fungal infection like mucormycosis, which was confirmed with a nasal swab on a sabourauds dextrose agar culture. Since diabetes is one of the most common comorbidities of mucormycosis, it is possible that the patient either had a previous undiagnosed mucor infection or it may have been aggravated with further dysregulation of immunity secondary to COVID-19. Use of steroids and monoclonal antibodies to treat COVID-19 may lead to exacerbation of opportunistic infections like mucormycosis as well^45^.

Pasero et al. (2020) discussed a case of a 66-year-old male patient with a past medical history of arterial hypertension, admitted to ICU in Italy with COVID-19. The patient was intubated due to COVID-19 complications. Over the course of 2 week, the patient had multiple organ dysfunction with sequential organ failure assessment (SOFA) score of 14, and his respiratory parameters continued worsening. Patient was started on empiric antibiotics therapy. After 2 weeks of empirical therapy a bronchial aspirate was repeated to confirm SARS-CoV-2 and to detect other coinfections. The bronchial aspirate confirmed coinfection of *Rhizopus spp.* and treated appropriately, yet the patient did not survive and died at day 62 of refractory shock and liver failure. The researchers here also suggested altered and impaired immune system were the cause of higher risk of opportunistic infection and negative outcome^5^.

Gago and Ibrahim (2021) represented a case of 53-year-old male patient with past medical history of obesity and depression was diagnosed with secondary acute myeloid leukemia (AML) in January 2020 and transferred to the hospital for further treatment. After five weeks of treatment the patient developed sore throat, paraguesia, dysosmia and fever and was diagnosed with SARS-CoV-2 confirmed with nasal RT-PCR at day 54 after induction of chemotherapy. On day 8 after SARS-CoV-2 diagnosis, patient required intubation, and was extubated on day 18 as he stabilized. However, shortly after he developed fever up to 39.5 °C with negative blood culture, acridine-orange leukocyte cytospin test, viral PCRs, and fungal biomarkers. Due to rapid respiratory deterioration patient required reintubation on day 22 with a positive SARS-CoV-2 nasopharyngeal swab and negative bronchoalveolar lavage. Due to worsening hemodynamic situation, the patient died on day 24. A full autopsy was performed and revealed invasive Rhizopus microspores from lung tissue. Researchers suggested chemotherapy combined with corticosteroids resulted in a prolonged neutropenic phase leading to opportunistic infection like mucormycosis. Due to negative testing of fungal infections the patient was not treated with any other azoles except voriconazole, an antifungal used a prophylaxis in such patients. This report was also intended to raise awareness for early detection and treatment of mucormycosis^46^.

Hanley et al. (2020) reported a post-mortem case series of nine patients with confirmed COVID-19 at premortem between March 1 and April 30, 2020. The median age at death of this cohort was 73 years (IQR 52-79). At least one major organ, predominantly the lung showed thrombotic features at full autopsies and diffuse alveolar damage was the most consistent lung finding in all ten patients. The series supported several novel autopsy findings, including secondary disseminated mucormycosis, requiring additional investigation to understand the role of this opportunistic infection in COVID-19 patients^47^.

Sen et al. (2021) conducted a retrospective, interventional study on 6 COVID-19 patients who developed rhino-orbital mucormycosis and were managed at a tertiary ophthalmic center in India between August 1 and December 15, 2020. All patients were men with mean age of 60.5 ± 12 with type 2 diabetes. All except one patient received systemic corticosteroids treatment for COVID-19. The researchers reported mean duration of 15.6 ± 9.6 days between diagnosis of COVID-19 and development of symptoms of mucor. All patients had undergone endoscopic sinus debridement and only two required orbital exenteration, however all six patients were alive at the last follow up. The researchers put much emphasis on high index of suspicion, early diagnosis, and appropriate management for patient survival^48^.

Kanwar et al. (2021) also reported a fatal case of 56-year-old man who was hospitalized for COVID-19. This patient had pre-existing end-stage renal disease on hemodialysis and ultimately developed mucormycosis during his hospitalization. Patient has a positive SARS-CoV-2 RT-PCR while asymptomatic, until four days later, he was hospitalized for fatigue and shortness of breath, at which time he received methylprednisolone, tocilizumab and single dose of convalescent plasma. Upon admission, blood cultures were negative for bacterial and fungal microorganisms. Patient was discharged home 7 days later, readmitted five days after discharge with generalized fatigue, shortness of breath and hemoptysis. Patient was started on empiric antibiotics therapy (IV vancomycin and piperacillin-tazobactam) for suspected healthcare-associated pneumonia. His chest x-ray showed increasing airspace density in both lungs with pleural effusion. On Day 3, a repeat sputum revealed filamentous fungus and empiric treatment of liposomal amphotericin B was started. Despite persistent drainage of pleural effusion with pigtail catheter over the next few days, repeat chest CT was unchanged. On repeated sample analysis, Rhizopus azygosporous was also diagnosed, and patient was put on appropriate treatment. Despite all efforts patient developed cardiac arrest and died on hospitalization day 17. Researchers recommended severe COVID-19 should be considered as a risk factor for invasive fungal infections, particularly those who receive immunosuppressive medications like steroids and IL-6 inhibitors (tocilizumab). Because there are no non-invasive tests available for invasive fungal infections, researchers believe cases of mucormycosis cases may be higher in numbers than what is reported^49^.

Moorthy et al. (2021) completed multi-centric retrospective study in Bangalore, India in 18 patients with diabetes mellitus (DM) with positive SARS-CoV-2 infections. 15 of 18 patients had confirmed uncontrolled DM and all received corticosteroid for COVID-19 treatment. Surprisingly, 12 of 18 patients had complained of vision loss, 7 of whom then underwent orbital exenteration. The results showed 16 cases of mucormycosis, 1 of aspergillosis and 1 case of mixed fungal infection. Six of these patients died, 11 survived and 1 was lost to follow up. Researchers confirmed significantly higher incidence of fungal infections (p = 0.03) amongst diabetic patients and suspect strong association with immunosuppression related to corticosteroid administration^50^.

Karimi-Galougahi et al. (2021) reported a case of 61-year-old woman with no past medical history and was hospitalized for COVID-19 infection for 2 weeks. During her admission, she received remdesivir, interferon-alpha, and systemic corticosteroid. Patient did not require intubation and mechanical ventilation. Patient developed right hemifacial pain with no other sinonasal symptoms, hemifacial numbness, decreased visual acuity, and chemosis one week after her discharge, which prompted her second hospitalization. The non-contrast CT of paranasal sinuses, MRI, and diagnostic sinonasal endoscopy suggested and confirmed invasive fungal infection of mucormycosis. In this case, although the patient was healthy, researchers indicated that the corticosteroid treatment induced diabetes and immunosuppression in addition to immune dysregulation caused by COVID-19 caused invasive mucormycosis, encouraging prompt diagnosis by physicians^51^.

Several other Rhino-orbital mucormycosis cases have been reported. Waizel-Haiat et al. (2021) reported a case of a 24-year-old female in Mexico City, with past medical history of obesity, who tested positive for COVID-19. She was brought to the emergency room, where she complained of left midface pain at least six days prior to admission, and within two days showed signs of left lid swelling and maxillary hypoesthesia. When oral amoxicillin-clavulanate did not provide relief, rhinoscopy was completed with contrast-enhanced CT of head and chest, revealing invasive fungal infection. This patient developed multiple other complications of COVID-19, including metabolic acidosis combined with pulmonary insult and acute kidney injury due to disseminated intravascular coagulopathy, leading to death due to multi-organic failure due to septic shock. The researchers concluded this patient had immunosuppressive state secondary to diabetic ketoacidosis, making her susceptible to COVID-19 and mucormycosis coinfections. Her late diagnosis and delay in treatment further contributed to this unfortunate outcome^52^.

Currently, there is only one ongoing clinical trial [NCT04368221] at Rennes University Hospital in France to assess prevalence of opportunistic fungal co-infections in COVID-19 infected and mechanically ventilated patients in ICU. Researchers feel ICU patients with ARDS are not systematically screened for the respiratory fungal infections. The study will allow to determine median time between entry in ICU and beginning of ARDS and colonization of fungal infections such as *Aspergillus, Pneumocystis jirovecii* and *mucoromycetes*. The study will also evaluate time between diagnosis and targeted treatment at ICU discharge, up to 1 month and further help develop preventive strategies^53^.

## 6. Conclusion

**Figure 1:**
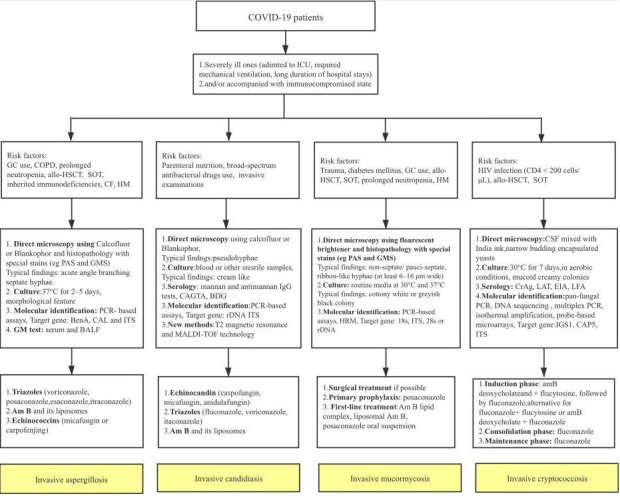
Diagnostic and therapeutic pathway for invasive fungal co-infections in COVID-19 patients **Reproduced with permission from reference^40^**.

**This report is intended to raise awareness for early detection and treatment of various fungal infections, particularly high mortality mucormycosis, in COVID-19 patients to reduce the risk of mortality. Due to association with a very high mortality rate, the researchers put much emphasis on high index of suspicion, early diagnosis, and appropriate management for patient survival. It is essential to assess the risk factors, the types of invasive mycosis to provide appropriate individualized treatment. Additional investigation is needed to understand the role of opportunistic infections in COVID-19 patients. Finally, we provide a flow diagram (Figure 1) to assist the clinicians and laboratory experts in the management of aspergillosis, candidiasis, mucormycosis, or cryptococcosis, as comorbidities in COVID-19 patients.
